# Treating repetitive suicidal intrusions using eye movements: study protocol for a multicenter randomized clinical trial

**DOI:** 10.1186/s12888-019-2129-0

**Published:** 2019-05-09

**Authors:** J. S. van Bentum, M. Sijbrandij, A. J. F. M. Kerkhof, A. Huisman, A. R. Arntz, E. A. Holmes, G. Franx, J. Mokkenstorm, M. J. H. Huibers

**Affiliations:** 1 Department of Clinical, Neuro- and Developmental Psychology, Amsterdam Public Health Research Institute, Vrije Universiteit Amsterdam, Van der Boechorststraat 7, 1081 BT Amsterdam, The Netherlands; 2 Department of Clinical Psychology, Universiteit van Amsterdam, Nieuwe Achtergracht 129, 1018 WS Amsterdam, The Netherlands; 3 Department of Psychology, University of Uppsala, Campus Blåsenhus, Von Kraemers allé 1A och 1C, Uppsala, Sweden; 4 Department of Implementation, 113 Suicide Prevention, Paasheuvelweg 25, 1105 BP Amsterdam, The Netherlands; 5 Department of Psychiatry, Amsterdam Public Health Research Institute, Vrije Universiteit Medical Center, De Boelelaan 1117, 1081 HV Amsterdam, The Netherlands; 6 Department of Research and Innovation, GGZ inGeest, Specialized Mental Health Care, Oldenaller 1, 1081 HJ Amsterdam, Netherlands; 7 Department of Research, 113 Suicide Prevention, Paasheuvelweg 25, 1105 BP Amsterdam, The Netherlands; 8 Department Psychology, University of Pennsylvania, Stephan A. Levin Building, 425 S. University Ave, Philadelphia, PA 19104-6018 USA

**Keywords:** Suicide, Suicidal intrusions, Mental imagery, EMDT, Dual tasks, Multicenter randomized controlled trial

## Abstract

**Background:**

Suicide is a major public health problem, and it remains unclear which processes link suicidal ideation and plans to the act of suicide. Growing evidence shows that the majority of suicidal patients diagnosed with major depression or bipolar disorder report repetitive suicide-related images and thoughts (suicidal intrusions). Various studies showed that vividness of negative as well as positive intrusive images may be reduced by dual task (e.g. eye movements) interventions taxing the working memory. We propose that a dual task intervention may also reduce frequency and intensity of suicidal imagery and may be crucial in preventing the transition from suicidal ideation and planning to actual suicidal behaviour. This study aims a) to evaluate the effectiveness of an Eye Movement Dual Task (EMDT) add-on intervention targeting suicidal imagery in depressed patients, b) to explore the role of potential moderators and mediators in explaining the effect of EMDT, and c) to evaluate the cost-effectiveness of EMDT.

**Methods:**

We will conduct a multi-center randomized clinical trial (RCT) evaluating the effects of EMDT in combination with usual care (*n* = 45) compared to usual care alone (n = 45). Participants will fill in multiple online batteries of self-report questionnaires as well as complete a semi-structured interview (Intrusion Interview), and online computer tasks. The primary outcome is the frequency and intrusiveness of suicidal imagery. Furthermore, the vividness, emotionality, and content of the suicidal intrusions are evaluated; secondary outcomes include: suicidal behaviour and suicidal ideation, severity of depression, psychological symptoms, rumination, and hopelessness. Finally, potential moderators and mediators are assessed.

**Discussion:**

If proven effective, EMDT can be added to regular treatment to reduce the frequency and vividness of suicidal imagery.

**Trial registration:**

The study has been registered on October 17th, 2018 at the Netherlands Trial Register, part of the Dutch Cochrane Centre (NTR7563).

## Background

Each year, close to 800,000 people worldwide die by suicide, and for every suicide there are many more people who attempt suicide [1]. While suicide prevention interventions have been developed, little remains known about which processes link suicidal plans to the act of suicide [2, 3]. However, one recent development in the suicide theory research is the ideation-to-action framework, which suggests that the development of suicidal ideation is distinct from the progression from ideation to suicide attempts [4]. One factor that may potentially be of influence on the latter, either directly or indirectly, is the presence of intrusive suicidal imagery (also called: suicidal intrusions). Various studies revealed that the majority of patients diagnosed with major depression or bipolar disorder report repetitive intrusive suicide-related images and thoughts [5–7]. These images may be both distressing and comforting, as well as compelling [8, 9]. Examples include images of their own suicide (jumping in front of a train, seeing themselves after an overdose) or the aftermath thereof (attending their own funeral, seeing the reaction of their family members).

Experimental and clinical studies showed that vividness of both negative and positive intrusive images may be reduced by dual task interventions taxing the working memory such as eye movements [10]. It is assumed that recalling an emotional image and performing eye movements both require working memory resources. Since the eye movements reduce resources for recall, the image is rendered less intense, even upon recall [11]. This may be a novel perspective on how suicidal intrusions may be addressed, so we have developed an Eye Movement Dual Task (EMDT) additive intervention. Currently, Eye Movement Desensitization and Reprocessing (EMDR), an evidence-based dual task-treatment for posttraumatic stress disorder (PTSD) that is characterized by trauma-related intrusions [12, 13] is also being applied in routine clinical practice to treat suicidal images. However, while EMDR has recently been applied effectively in treating other psychiatric disorders such as psychosis [14], there is no empirical evidence yet that this technique is effective for suicidal intrusions.

The relevance of our study is that, if proven effective, the addition of EMDT to regular treatment may reduce the frequency and vividness of suicidal imagery. This paper describes the trial procedures for this study. This multicenter randomized trial aims to evaluate the effectiveness of EMDT in reducing suicidal mental imagery in depressed patients. In addition, we will evaluate the potential mediating effect of suicidal imagery on suicidal ideation, as well as depressive symptoms. Next, we will explore the potential role of the following moderators and mediators in explaining the effect of EMDT itself: a reduction in suicidal ideation, baseline depressive symptom severity, presence of childhood trauma, obsessive-compulsive traits, and potential effects of taxing the working memory. Lastly, we will evaluate the cost-effectiveness of EMDT.

## Methods

### Study design

**Fig. 1:**
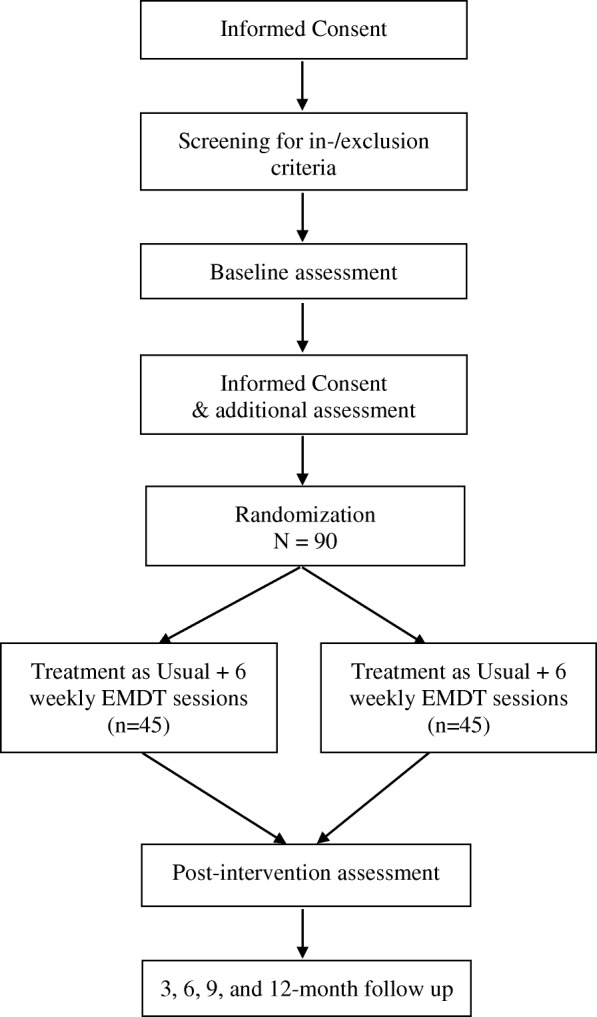
Flow diagram

The study is a multicenter randomized clinical two-armed, parallel-designed trial that will evaluate the effects of EMDT in combination with treatment as usual (TAU) compared to TAU alone. The anticipated flow of subject enrolment is graphically shown in Fig. 1. The primary outcome is the frequency and intrusiveness of suicidal imagery. Furthermore, the vividness, emotionality, and content of suicidal intrusions are evaluated. Secondary outcomes are suicidal ideation, suicidal behaviour, severity of depression, rumination, and hopelessness. Several candidate moderators and mediators of outcome are also assessed alongside the outcome variables. We are looking at variables such as: rumination (Ruminative Response Scale, RRS; [15]), childhood trauma (Childhood Trauma Questionnaire, CTQ-SF; [16]), obsessive-compulsive traits (Obsessive-Compulsive Inventory, OCI-R; [17]), intrusions (Response to Intrusions Questionnaire, RIQ and Breathing Focus Task, BFT; [18, 19]), and imagery (Prospective Imagery Task, PIT; [20]). This study protocol followed SPIRIT guidelines and fulfilled the SPIRIT checklist.

### Ethics and data safety monitoring board (DSMB)

The Medical Ethics Committee of the VU Medical Center approved the study protocol (registration number 2017.237), after we conducted a small pilot study (*n* = 6) to establish the safety and feasibility of the intervention [21]. The study is registered at the Netherlands Trial Register, part of the Dutch Cochrane Centre (NTR7563). Furthermore, an independent DSMB has been established to assess the progress, the safety data, and the critical efficacy endpoints of the study. The DSMB will provide recommendations to the principal investigator and associated project group members about the safety, the study conduct, and the scientific validity and data integrity of the study. Its tasks involve: evaluating the accumulating safety assessments to ensure the ongoing safety of study subjects, and monitoring the conduct of the study including any protocol violations.

### Participants

We aim to include 90 participants that satisfy the following criteria: a. score ≥ 1 on the Suicidal Ideation Attributes Scale (SIDAS; [22]); b. score ≥ 20 on Beck’s Depression Inventory II (BDI-II; [23]); c. age ≥ 18; d. adequate proficiency in the Dutch language; e. currently receiving treatment as usual (TAU) at a mental health institution; and f. have suicidal intrusions that are experienced as a burden. The participant must answer positive on one of the following questions: ‘do the suicidal intrusions hinder you in your daily life?’ and ‘do you feel tormented by these suicidal intrusions?’

Patients will be excluded when a. they are diagnosed with at least one of the following disorders: DSM-IV psychotic disorder, DSM-IV depression with psychotic features, or DSM-IV bipolar disorder; b. they have no access to internet; and c. there is high dropout risk (i.e. poor response rate when trying to get in contact with potential participant). The presence of DSM-IV disorders will be indicated by a diagnosis following the Mini International Neuropsychiatric Interview [24].

### Sample size

We cannot provide estimates on the reduction of suicidal intrusions as a result of the study, as we are not aware of similar interventions tested in this population and the outcome measure (Intrusion Interview; [6]) does not produce a standardized outcome. Therefore, we based our power analysis on the expectation of a medium to large effect size of 0.7 of TAU + EMDT as compared to TAU at 3 months on the primary outcome (suicidal intrusions). Power calculations suggest a minimum sample size of 38 participants per group (power = 0.85, alpha = 0.05, two-sided). Accounting for 15–20% attrition, we aim to include 45 participants per group. This adds to a total of 90 participants.

### Procedure

The study will be carried out at various Dutch specialized mental healthcare centers including Arkin (Amsterdam), Altrecht (Utrecht), Dimence (Zwolle), GGZ Oost-Brabant (Oss, Boekel, Helmond, Boxmeer), GGZ Eindhoven, Parnassia Groep (The Hague), Pro Persona (Nijmegen), and the Vincent van Gogh Institute (Venray, Venlo). Each healthcare center will have its own contact person/research assistant, and patients will be recruited within the appropriate department.

After providing oral and written informed consent, patients will be screened for suicidal ideation (SIDAS), depressive symptoms (BDI-II) and DSM-IV diagnoses (M.I.N.I.). Next, the breathing focus task (BFT) is administered and participants are interviewed (using the Intrusion Interview) by trained staff members of the mental health care institution where they are currently being treated. They are asked to complete a set of (online) self-report instruments and answer the following screening questions: ‘do the suicidal intrusions hinder you in your daily life?’ and ‘do you feel tormented by these suicidal intrusions?’. If they answer yes on at least one of those statements, they are invited for the next appointment. During this appointment, they will provide oral and written informed consent and the schematic overview of the Intrusion Interview is administered. While at home, they are asked to fill in additional questionnaires and perform a computer task (n-back task). Next, the participant is randomized into the intervention group (EMDT + TAU) or the control group (TAU only). Participants in the experimental group will, in addition to their TAU, receive one introduction meeting with an EMDT-therapist, a maximum of six weekly EMDT add-on sessions, and six weekly online assessments (SIDAS, BDI-II). Participants in the control group will, in addition to their TAU, receive the same six weekly online assessments. Additional assessments will be carried out at: pre-treatment (baseline), during treatment, post-treatment (six weeks after baseline), and at 3, 6, 9 and 12 months follow-up.

During our pilot study, we had evaluated whether the amount of (online) questionnaires was reasonable, and from participants’ feedback we concluded that it was. The self-report assessments will be administered online following standardized time points. Patients who are screened for eligibility but who are not burdened by suicidal intrusions and therefore excluded from trial participation will still be asked to complete an online 12-month follow up assessment. This follow up assessment may provide us with some insight on the potential symptom changes in participants that don’t experience suicidal intrusions as a burden.

### Randomization and blinding

Randomization will occur following the pre-assessment/baseline assessment. Participants will be randomized on a 1:1 basis (stratified for mental health care institution) into the intervention group (EMDT + TAU) or the control group (TAU). Randomization will be conducted using the stratified block randomization module in an electronic data capture system (Castor EDC). Participants randomized in the EMDT intervention will be allocated to an EMDT therapist at the participating mental health institution. The EMDT therapist will plan six consecutive meetings with the participant. Outcome assessors will be blinded when administering the primary outcome, the table in the appendix of the Intrusion Interview. The participant is informed that the outcome assessor is blinded, and is therefore asked not to disclose whether they have received the EMDT intervention or not.

### Eye movement dual task (EMDT)

The EMDT protocol has been developed in co-creation with patients, practitioners, and experts in the field of EMDR and suicidality, and builds on earlier work in the field of dual tasks [25]. The input of seven (formerly) depressed patients with suicidal ideation was obtained through a focus group. The goal was to provide insight into experiences and ideas of people who had to deal with depression and suicidality in the past. Questions regarding their experiences with suicidal intrusions, treatment for suicidality, and their opinions about the EMDT additive intervention were discussed. In addition, an expert review round was held, in which six different national experts shared comments and suggestions to further optimize the protocol by means of their knowledge. The intervention will be an add-on module that addresses intrusive suicidal images and will run parallel to regular treatment. The EMDT sessions include a dual task component and try and reduce the frequency, vividness, and emotionality of the suicidal intrusions (desensitization). A negative cognition is identified to address the associations of the intrusions. In the well-known EMDR therapy, a positive cognition is also linked to the image [25]. The purpose of installing this positive line of thought is to maximize the credibility of this positive cognition. Since our goal is to reduce vividness and emotionality and therefore frequency of the intrusions, the ‘installation’ process is not applicable.

The intervention will consist of a maximum of six sessions each of approximately 1 h, in the course of six weeks, delivered at the patients’ mental health care center. Each session will consist of the following steps:

1. The patient and EMDT therapist discuss and determine which suicidal intrusion will be treated this session. This could either be a) a new suicidal intrusion, b) the intrusion of the last session that needs continued work or c) a last check of an intrusion that ended with a score of 0 on the Subjective Units of Distress Scale (SUDS, scale 0–10) in the previous EMDT session. The SUDS is administered to assess the level of distress during image retrieval. In addition to a clear target image, the associated negative cognition and emotions are put in focus.
2. The patient performs consecutive set of eye movements of 30 s by 10 s breaks. Between the sets, the SUDS is administered.
3. If the image still produces stress, the dual task procedure will be repeated for the target image.
4. This procedure is repeated for all target images until all SUDS are at approximately 0 or no longer diminish.

At the end of each session, the therapist and patient briefly evaluate the most positive or worthwhile part of the session. This is to make sure that the session is not abruptly ended at a high emotional point. Moreover, the therapist and patient create a self-care plan for the rest of the day. All sessions will be recorded on videotape, but only after consent from the participant.

Therapists carrying out the EMDT intervention have successfully completed a 3,5 to 4-h training, in which suicidal intrusions and the EMDT research protocol were discussed in great detail. In addition, the training included practice rounds. Each EMDT therapist receives supervision sessions per participant from an appointed member of the research project. All EMDT therapists are employed at the participating mental health centers.

### Treatment as usual (TAU)

TAU for depression and other psychological disorders within the participating mental health care institutions typically consists of (evidence-based) psychotherapy and/or antidepressant treatment. We will ensure that all patients will receive and continue TAU during the course of the study. After each assessment the TAU mental health care provider will be updated on the progress of the patient, with consent of the patient.

### Safety regulations

Using the results from our small mono-center pilot study, we have created a safety protocol. The purpose is to provide a brief summary of additional requirements that have to be met surrounding the EMDT intervention, to ensure as much safety as possible. It is designed to be a supplement to the EMDT research protocol. The safety protocol states that each participant should have an obligatory safety plan. Furthermore, suicidal ideation is screened during the EMDT and if SIDAS scores reach above a certain cut-off score (> 25), the therapist may be notified. There is an explicit focus on the question: ‘in the past week, how close were you to a suicide attempt?’ (ranging from 0 = not close at all to 10 = I have made a suicide attempt).

### Instruments

An overview of all patient instruments per assessment is given in Table 1. The duration of the (online) questionnaires range from 10 to 70 min.

**Table 1:** Overview of patient instruments per time point

Instruments	S	B	T1	T2	T3	3	6	9	12
Suicidal Intrusions									
Intrusion Interview (II)		•	•		•	•	•	•	•
Response to Intrusions Questionnaire (RIQ)		•							
Suicidal Intrusions Attributes Scale (SINAS)		•			•	•	•	•	•
Suicidal Ideation									
Suicidal Ideation Attributes Scale (SIDAS)	•	•		•	•	•	•	•	•
Beck Scale for Suicidal Ideation (BSSI)		•			•	•	•	•	•
Depression									
Beck Depression Inventory (BDI-II)	•	•		•	•	•	•	•	•
Quality of life									•
EQ-5D-5L			•				•	•	•
Societal Costs									
Trimbos/iMTA questionnaire for Costs associated with Psychiatric Illness (TiC-P)			•			•			•
Psychological Problems									
Brief Symptom Inventory (BSI)		•			•	•			•
Rumination									
Ruminative Response Scale (RRS-NL)		•			•	•			•
Obsessive Compulsive Disorder									
Obsessive Compulsive Inventory – Revised (OCI-R)		•			•	•			•
Hopelessness									
Beck Hopelessness Scale (BHS)		•			•	•			•
Childhood trauma									
Childhood Trauma Questionnaire (CTQ-SF)		•							
Other measures									
M.I.N.I. Neuropsychiatric Interview	•								
Demographics Questionnaire		•							
N-back Task			•		•				
Breathing Focus Task (BFT)		•							
Prospective Imagery Task (PIT)		•							
Care-as-usual Treatment Details					•				
EMDT Treatment measures			•		•				

*S* Screening, *B* Baseline assessment, *T1* Pre-treatment assessment, *T2* During treatment, *T3* Post-treatment, *3* 3 month follow-up, *6* 6 month follow-up, *9* 9 month follow-up, *12* 12 month follow-up

#### Intrusion interview

There are currently no formally validated measures available to assess the content and frequency of suicidal intrusions. We will use the most well-established measure available. The semi-structured Intrusion Interview is a translated (and adapted) version of the English Suicidal Cognitions and Flashforwards Interview [6] and assesses the content of mental images about suicide and verbal thoughts. The interview consists of 21 items, including a 9-item checklist used to assess and describe in detail the content of cognitions of the most significant intrusion patients experience when they are most despaired. To obtain a clear schematic overview of the frequency and intensity of the current suicidal intrusions experienced by the participant, we slightly adapted the Intrusion Interview by adding a table as an appendix. This table is our primary outcome and measures the frequency, vividness, intrusiveness, and uncontrollability of suicidal intrusions. Patients are asked to recall and report the frequency of intrusions experienced per day for the past week, and rate image distress and burdensomeness on 10-point scales (0 = not at all to 10 = extremely). To clarify, the table is filled in retrospectively with the trained staff member while administering the Intrusion Interview. The schematic overview explicitly focuses on mental images and is based on an intrusion diary [26].

#### Suicidal ideation attributes scale (SIDAS; [22])

The SIDAS is a self-report instrument measuring the presence and severity of suicidal ideation, and can be administered electronically. It contains five items assessing the frequency, controllability, closeness to attempt, distress, and interference with daily activities on 10-point scales over the past month. Total scores are calculated as the sum of the five items, with controllability reverse scored and range between 0 and 50. Scores above 21 indicate a high risk of suicidal behavior. The SIDAS demonstrated high internal consistency (Cronbach’s a 0.91–0.86) and takes between 30 and 60 s to complete.

#### Beck depression inventory (BDI-II; [23])

The BDI-II is a well established measure of depression and has shown excellent reliability and validity (Dutch version: [27]). It contains 21 items, comprising of four self-evaluative statements about a particular symptom of depression, scored 0 to 3, with increasing scores indicating greater depression severity. Total scores range between 0 and 63. Scores above 16 indicate a clinical depression. The time frame of the BDI-II questions comprises two weeks. Reliability and validity of the Dutch version of the BDI-II has been well established [27, 28]. High internal consistency in a clinical population was found (Cronbach’s a 0.92).

#### Mini international neuropsychiatric interview (M.I.N.I.; [24])

The M.I.N.I. is a short structured psychiatric interview used to assess current and past diagnostic status of DSM-IV disorders. It takes about 15 min to administer. Comparisons of the M.I.N.I. with the Structured Clinical Interview for DSM-II-R (SCID), Composite International Diagnostic Interview (CIDI), Diagnostic Interview Schedule (DIS), and Present Status Examination (PSE) showed that the M.I.N.I. has reasonably high validity and reliability [29].

#### Suicidal intrusions attributes scale (SINAS; [30])

The SINAS is a new scale we developed for the purpose of this study [30]. It is based on the SIDAS and measures the characteristics of suicidal intrusions in the past week. It includes 10 items asking about the frequency, intensity, vividness, and uncontrollability of the intrusions. Each item is scored on a 10-point scale (e.g. ‘how often did you experience images about your own suicide’ is scored as 0 = not at all to 10 = constantly), and the total score ranges between 0 and 100.

#### Beck scale for suicide ideation (BSSI; [31])

The BSSI is a self-report instrument that contains 19 items assessing an individual’s beliefs and attitudes about suicide such as frequency and duration of ideation, specificity of planning, and preparations for death for the past week (including the day of assessment). Each item has three options, which are rated on a 3-point scale from 0 to 2, a higher score indicating a higher level of suicidality. The total score of the BSSI ranges from 0 to 37, with higher scores indicating more severe suicidal ideation. The BSSI has very good internal consistency and convergent validity, and has been found to predict future suicide attempts and death by suicide [32]. A validated Dutch version was created [33].

#### EQ-5D-5 L [34]

The EQ-5D-5 L is used to assess quality of life and enables a rudimentary economic evaluation of the effects of the EMDT add-on intervention, weighing costs and effects. It is a generic instrument for describing and valuing health and is based on a descriptive system that defines health in terms of five dimensions: mobility, self-care, usual activities, pain/discomfort, and anxiety/depression. Respondents are asked to indicate their health state by choosing the most appropriate statement in each of the five dimensions. This results in a 1-digit number expressing the level selected for that dimension. In turn, the digits can be combined in a 5-digit number describing the respondents’ health state.

#### Trimbos/iMTA questionnaire for costs associated with psychiatric illness (TiC-P; [35])

The societal costs during this study will be measured using the TiC-P, which consists of two parts. The first part focuses on the use of (psychiatric) health care including primary and secondary care, complementary care and home care (4 items). The second part focuses on compromising costs due to loss of production including absenteeism from paid and unpaid work, and presenteeism (15 items). The valuation of health care utilization standard prices will be used [36], and medication use will be valued using prices of the Royal Dutch Society for Pharmacy (Z-Index, 2006).

#### Brief symptom inventory (BSI; [37])

The BSI is a 53-item self-report questionnaire that administers a multidimensional complaint list that shows the extent to which a participant suffers from various psychological and physical symptoms. Each item is rated on a 4-point scale (0 = none at all to 4 = a lot) and are divided into nine subscales: somatic complaints, cognitive problems, interpersonal sensitivity, depressed mood, fear, hostility, phobic fear, paranoid thoughts, and psychoticism. A version has been translated to Dutch by de Beurs and Zitman [38].

#### Obsessive-compulsive inventory – revised (OCI-R short version; [17])

The OCI-R is a short version of the original OCI and is a self-report scale for assessing symptoms of Obsessive-Compulsive Disorder (OCD). It contains 19 questions that the participant answers using a 5-point Likert scale. The item scores are added up and the possible range of scores is between 0 and 72. Scores 21 or above indicate the likely presence of OCD. A version has been translated to Dutch by Cordova-Middelbrink, Dek and Engelbarts [39].

#### Ruminative response scale (RRS; [15])

The RRS is a self-report measure that assesses ruminative responses to depressed mood. It is a revision of the original longer Response Styles Questionnaire. It consists of 22 items and measures two aspects of rumination: brooding and reflective pondering. Each item is rated on a 4-point scale (1 = almost never to 4 = almost always), and the possible range of scores is between 22 and 88. The Dutch translation of the scale has been found to be internally consistent (a = .90; [40]).

#### Beck hopelessness scale (BHS; [41])

The BHS is a widely used standardized measure of hopelessness. The BHS consists of 20 ‘true-false’ statements covering positive and negative thoughts about the true-false response. The item scores are added up and the possible range of scores is between 0 and 20. The predictive validity of the BHS for suicide and attempted suicide was supported by the authors of the scale [42], and was confirmed by later research [43, 44]. The Dutch translation of the scale has been found to be internally consistent (a = 0.68–0.75; [45]).

#### Childhood trauma questionnaire-short form (CTQ-SF; [16])

The CTQ-SF is a 28-item retrospective self-report questionnaire that is designed to assess five types of negative childhood experiences: emotional neglect, emotional abuse, physical neglect, physical abuse, and sexual abuse. The tendencies to minimize or deny abuse experiences are measured as well. The truth of each statement is rated on a 5-point scale. The CTQ-SF has adequate reliability and validity [16].

#### N-back task [46]

The N-back task is used to measure executive functioning. Patients are asked if the letter on the screen matches a letter previously (0 (baseline), 1, 2, or 3 back) presented. Only when the patient performs well on the 2-back, will he or she be forwarded to the 3-back part of the task. Working memory load increases as the task progresses from 1-back to 3-back and is suggested to require executive processes. The task will take around 15 min and the accuracy of responses and reaction times will be measured.

#### Breathing focus task (BFT; [19])

The BFT is a measure of the behavioural state related to intrusive thoughts. It consists of 5 min of focus on breathing, where patients are asked 12 times about their current thoughts. Patients briefly describe the current thought in keywords and indicate the emotionality (positive, neutral, negative). After the 5-min breathing focus phase, each of the none-breathing answers (so each intrusion) is discussed with the patient to get more insight on the nature of the thought.

#### Prospective imagery task (PIT; [47, 48])

Participants are asked to form a mental image of 10 negative future scenarios and 10 positive future scenarios. These include events such as: ‘you will have a serious disagreement with your friend’ or ‘you will do well on your course’. Images are rated on vividness on a 5-point Likert scale (ranging from 1 = no image at all to 5 = very vivid). The PIT has good internal consistency [47]. This task can and will be administered as a questionnaire.

#### Response to intrusions questionnaire – negative appraisal subscale (RIQ; [18, 49])

Participants are asked about a negative memory they experienced in the past week. Six items assessing negative appraisals of intrusions are rated on a 7-point Likert scale (ranging from 1 = totally disagree to 7 = totally agree). This subscale is reported to have good internal consistency (a = 0.84; [49])*.*

#### EMDT treatment measures

Various aspects of the intervention preceding and following the EMDT sessions will be administered. Participants will answer six questions about their expectations of the treatment (e.g. thoughts and feelings about the efficacy of the treatment and expectations about recovery). These questions will be answered before being randomized. EMDT-treatment compliance will be operationalized as the amount of no-shows (not showing up without notifying the EMDT therapist), cancelled and replaced appointments. Patients in the intervention group will answer 19 items evaluating the EMDT add-on sessions. Patients are asked to rate aspects of the treatment on a ten-point scale (ranging from 0 = not good at all to 10 = very good) and provide feedback and possible suggestions. An explanation is asked (5 items) why the patient thinks his or her symptoms did (not) improve.

#### Treatment integrity

In order to measure therapist adherence to the EMDT treatment protocol, independent raters will review a random selection of videotaped EMDT sessions. For the purpose of this study, we have developed an EMDT-session Checklist that describes the essential parts of each session. The independent raters will use this 12-item dichotomous yes/no checklist, to check therapist adherence to the treatment condition.

### Data analyses

Data-analyses will include intention-to-treat analysis and additional subgroup analyses. Missing data will be imputed using multiple imputation. The reduction in frequency of intrusive suicidal imagery is analyzed using hierarchical linear modeling. Between-group scores on secondary measures (like BDI, SIDAS, BSSI, RRS, and BHS) will also be compared using hierarchical linear modeling.

Latent difference score (LDS) models are used to examine any potential mediation effects of suicidal imagery on suicidal ideation, depression, and hopelessness. LDS models will also be used to investigate a potential underlying mechanism of the intervention itself using the pre- and post-treatment scores on the n-back task. Moderators may play a direct role in elaborating mediators and mechanisms of change [50, 51], thus we are looking at potential (moderated) mediation effects using potential moderating variables such as childhood trauma, rumination, and obsessive-compulsive traits. To examine potential mechanisms of change, both multilevel models and structural equation models (SEM) will be used.

#### Cost-effectiveness analyses

To analyze the cost-effectiveness of the EMDT add-on treatment, societal costs will be measured using the TiC-P at baseline and after 3, 6, 9, and 12 months. Direct societal costs entail primary and secondary care, complementary and home care. Indirect costs entail absenteeism from paid and unpaid work, and presenteeism. The latter costs will be determined using a friction cost approach. Costs will be related to the following effect measures in the economic evaluation: the decrease in frequency and vividness of suicidal imagery as measured through the schematic overview in the appendix of the Intrusion Interview, and Quality-adjusted-life-years (QALYs) based on the Dutch tariff for the EuroQol (EQ-5D-5L).

### Data management

The proceedings of data security and storage are established in a data management plan. This plan was written according to the guidelines of the funding agent ZonMw. For detailed information about this plan, please contact the first author.

## Discussion

We presented a study protocol to examine the effectiveness and cost-effectiveness of an EMDT intervention added to regular treatment to reduce the frequency and intrusiveness of suicidal intrusions. This study will be the first to investigate experimental effects of treating suicidal intrusions with a dual task method. Given that prior to a suicide attempt, individuals often have passed through various stages including suicidal ideation, and the presence of these uncontrollable, vivid suicidal intrusions could potentially have an influence on the transition from suicidal ideation to actual suicidal behaviour. The EMDT add-on intervention, if proven effective, may therefore be an innovative way to directly reduce the frequency and burden of suicidal imagery [52]. We hope that our study will pave the way for larger studies to evaluate the effectiveness of EMDT on prevention of transition from suicidal ideation to suicidal behavior and on reducing the risk for suicidal behaviour.

## Study status

This is an ongoing study, and the investigators are currently collecting data. Thus far, we have recruited seven mental health institutions that are willing to participate. Each mental health institution has at least one team/department in which EMDT therapists are trained and patients screened for potential inclusion.

## Abbreviations

BDI-II: Beck Depression Inventory
BFT: Breathing Focus Task
BSSI: Beck Scale for Suicidal Ideation
CTQ-SF: Childhood trauma questionnaire – short form
DSMB: Data safety monitoring board
DSM-IV: Diagnostic and statistical manual of mental disorders – 4th edition
EMDR: Eye movement and desensitization reprocessing
EMDT: Eye Movement Dual Task
LDS: Latent difference scores
M.I.N.I.: Mini international neuropsychiatric interview
NTR: Netherlands Trial Register
OCI-R: Obsessive compulsive inventory – short version
PIT: Prospective Imagery Task
PTSD: Posttraumatic stress disorder
QALYs: Quality-adjusted-life-years
RCT: Randomised controlled trial
RIQ: Response to Intrusions Questionnaire
RRS: Ruminative Response Scale
SIDAS: Suicidal Ideation Attributes Scale
SINAS: Suicidal Intrusions Attributes Scale
SUDS: Subjective Units of Distress Scale
TAU: Treatment as Usual
TiC-P: Trimbos/iMTA questionnaire for Costs associated with Psychiatric Illness
